# Repurposed drug screen identifies cardiac glycosides as inhibitors of TGF-β-induced cancer-associated fibroblast differentiation

**DOI:** 10.18632/oncotarget.8609

**Published:** 2016-04-06

**Authors:** David T. Coleman, Alana L. Gray, Charles A. Stephens, Matthew L. Scott, James A. Cardelli

**Affiliations:** ^1^ Louisiana State University Health Sciences Center, Feist-Weiller Cancer Center, Shreveport, LA, USA

**Keywords:** cancer-associated fibroblast, cardiac glycosides, tumor microenvironment, drug screen, digoxin

## Abstract

The tumor microenvironment, primarily composed of myofibroblasts, directly influences the progression of solid tumors. Through secretion of growth factors, extracellular matrix deposition, and contractile mechanotransduction, myofibroblasts, or cancer-associated fibroblasts (CAFs), support angiogenesis and cancer cell invasion and metastasis. The differentiation of fibroblasts to CAFs is primarily induced by TGF-β from cancer cells. To discover agents capable of blocking CAF differentiation, we developed a high content immunofluorescence-based assay to screen repurposed chemical libraries utilizing fibronectin expression as an initial CAF marker. Screening of the Prestwick chemical library and NIH Clinical Collection repurposed drug library, totaling over 1700 compounds, identified cardiac glycosides as particularly potent CAF blocking agents. Cardiac glycosides are traditionally used to regulate intracellular calcium by inhibiting the Na^+^/K^+^ ATPase to control cardiac contractility. Herein, we report that multiple cardiac glycoside compounds, including digoxin, are able to inhibit TGF-β-induced fibronectin expression at low nanomolar concentrations without undesirable cell toxicity. We found this inhibition to hold true for multiple fibroblast cell lines. Using real-time qPCR, we determined that digoxin prevented induction of multiple CAF markers. Furthermore, we report that digoxin is able to prevent TGF-β-induced fibroblast contraction of extracellular matrix, a major phenotypic consequence of CAF differentiation. Assessing the mechanism of inhibition, we found digoxin reduced SMAD promoter activity downstream of TGF-β, and we provide data that the effect is through inhibition of its known target, the Na^+^/K^+^ ATPase. These findings support a critical role for calcium signaling during CAF differentiation and highlight a novel, repurposable modality for cancer therapy.

## INTRODUCTION

Fibroblasts are known to contribute to the development and progression of cancers as well as fibrotic diseases such as pulmonary fibrosis, scleroderma, and chronic kidney disease [1, 2]. Tumor progression is accompanied by the activation of stromal fibroblasts into myofibroblasts, often termed cancer-associated fibroblasts (CAFs) [3–6]. CAFs are characterized by increased expression of cancer-driving growth factors, including hepatocyte growth factor (HGF) and vascular endothelial growth factor; extracellular matrix (ECM) components, such as fibronectin, collagen, and tenascin C; and enhanced contractility that influences cancer cells through mechanotransduction and ECM remodeling [1, 7]. It is well documented that the cumulative effect of this CAF phenotype in the reactive stroma can be the promotion of invasive and metastatic cancer [8]. It has been shown that the presence of CAFs in the reactive stoma is correlated with poor prognosis for cancers of multiple origins [9, 10]. Multiple studies using genetically altered fibroblasts in mouse models support a role for the CAF phenotype in cancer initiation and progression. For example, mouse fibroblasts overexpressing HGF were able to initiate cancer in implanted normal human breast epithelia [11]. MCF7 cells were shown to grow into larger tumors when co-injected into mice with CAFs vs. normal fibroblasts [12]. Similarly, otherwise noninvasive cancer cells become invasive when co-injected into mice with CAFs [13].

Under normal physiological conditions feedback mechanisms restrict myofibroblast activation; however, in cancer and other fibrotic diseases this process can become dysregulated, leading to constitutive activation [1, 2, 14]. Overabundance of the potent morphogenic growth factor, transforming growth factor-β (TGF-β), in the tumor microenvironment is a primary contributor to aberrant CAF activation [15–19]. Binding of TGF-β to its receptor on fibroblasts initiates signaling cascades including dimerization and translocation of SMAD transcription factors into the nucleus [20]. SMAD promoter activity directly and indirectly regulates much of the CAF-specific gene expression [21].

Given the impactful role of CAF in cancer progression and fibrotic disease we sought to discover clinically relevant agents able to block TGF-β-induced CAF differentiation. Although some reports indicate complete depletion of myofibroblasts from certain tumors may worsen prognosis in mice, the authors acknowledge, and we agree, selective inhibition of CAF differentiation may be more efficacious by supporting normal tissue architecture maintained by quiescent fibroblasts [22]. To this end, we developed a high content immunofluorescence imaging assay to screen for compounds able to block the induction of fibronectin expression by TGF-β in fibroblast cell lines. Two drug libraries, the NIH Clinical Collection and the Prestwick Chemical Library were screened for efficacy. These libraries comprise over 1700 compounds previously approved for safety by the FDA (Prestwick) for at least one indication or at least used in clinical trials with pharmacology and toxicity information. Ideally, hits from these repurpose collections hold the potential of expedited approval for future clinical trials [23–28].

Herein we report, for the first time, the identification of cardiac glycosides, Na^+^/K^+^ ATPase inhibitors used to treat congestive heart failure, as potent inhibitors of TGF-β-induced CAF differentiation [29]. Results from our compound screen highlighted this class of compounds as hits. Secondary testing of the glycoside digoxin using western blot analysis, real-time qPCR, and ECM contractility assays confirmed the ability of digoxin to prevent CAF differentiation in multiple fibroblast lines without causing cell death. The effects of digoxin corresponded to its ability to reduce SMAD promoter activity and suggest a critical role for intracellular calcium signaling in CAF differentiation. These findings support further evaluation of cardiac glycosides and like compounds as therapeutic modalities for restricting myofibroblast activity in the cancer microenvironment and additional fibrotic diseases.

## RESULTS

### Cardiac glycosides identified as inhibitors of fibronectin expression in a screen of repurposed drug libraries

**Figure 1:**
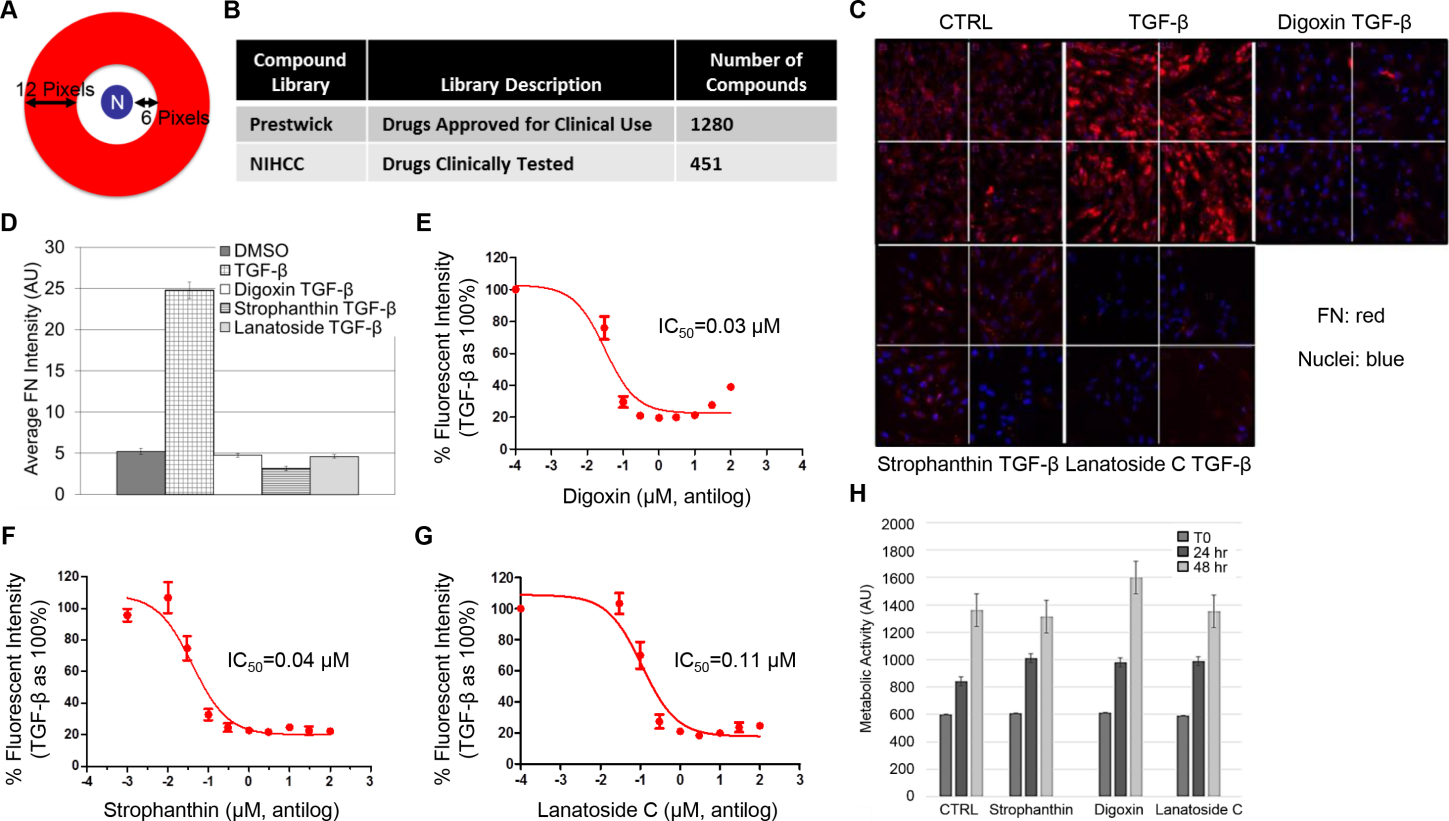
High-content screening reveals sub-toxic concentrations of cardiac glycosides inhibit TGF-β-induced fibronectin expression (**A**) Schematic illustrating the algorithm designed to analyze fluorescence intensity within a ring from 6 to 12 pixels (red) surrounding each nucleus (N). (**B**) Table documenting repurposed drug libraries with description and number of compounds screened from each. (**C**, **D**) WPMY-1 cells were serum starved for 24 hours followed by an additional 24 hours with TGF-β alone or TGF-β with 10 μM of the indicated compounds from the drug libraries. Cells were immunostained for fibronectin and imaged at 10× magnification using the Cellomics high-content imaging system (C). Average fluorescence intensity (arbitrary units, AU) was quantitated per cell based on DAPI staining (D). (**E**) The Cellomics-based fibronectin assay was repeated with or without TGF-β ± increasing concentrations (.001– 100 μM) of hit compounds: digoxin (E), strophanthin (**F**), and lanatoside C (**G**). To determine IC50 for each, results were plotted as fluorescence intensity relative to TGF-β set to 100%. (**H**) WPMY-1 cells were treated with strophanthin, digoxin, or lanatoside C at their respective IC50 for 0, 24, or 48 hours. Metabolic activity (arbitrary units, AU) was determined by the Cell Titer Blue Assay to indicate cell viability.

In order to discover compounds able to prevent CAF differentiation, we developed a high content immunofluorescence screen using the Cellomics Imaging Platform. The WPMY-1 fibroblast cell line was used for the initial screen. WPMY-1 cells were derived from the peripheral zone of a histologically normal prostate [30]. These cells upregulate fibronectin, and other CAF markers, in response to overnight treatment with TGF-β (Entrez Gene: 7040) [19]. For the screen, WPMY-1 cells arrayed in 96-well plates were treated with TGF-β in the presence or absence of test compounds at 10 μM for 24 hours under serum-free conditions. Changes to fibronectin expression were quantitated based on endpoint immunostain intensity per nuclei using a custom Cellomics software algorithm (Figure 1A). The screen incorporated 1280 compounds from the Prestwick Chemical library and 451 compounds from the NIH clinical collection (Figure 1B). Of the roughly 1700 compounds tested, the cardiac glycoside class of compounds (8 members; patent pending #62/290,002) was found to be consistently and potently effective at blocking TGF-β-induced fibronectin expression (Figure 1C, 1D).

The Cellomics imaging platform and algorithm were then used to establish a dose response curve and IC_50_ values for the most effective glycosides. Again, WPMY-1 cells were treated with TGF-β in the presence or absence of the cardiac glycosides at concentrations ranging from 1 nM-100 μM for 24 hours under serum-free conditions. The IC_50_values for digoxin, strophanthin, and lanatoside C were determined to be 30 nM, 40 nM, and 110 nM respectively (Figure 1E–1G). Viability assays were subsequently performed to address whether the compounds were blocking fibronectin induction or causing a toxic response. WPMY-1 cells were treated with digoxin, strophanthin, or lanatoside C at their respective IC_50_ and assayed for NADPH-based metabolic activity as an indication of cell viability. No impairment to metabolic activity was detected with any of the three glycosides through 48 hours of treatment (Figure 1H). These results suggest cardiac glycosides block TGF-β-induced fibronectin induction in WPMY-1 cells without causing appreciable cell death. Digoxin was used for subsequent studies to further determine whether cardiac glycosides were able to prevent the differentiation of fibroblasts to CAFs.

### Digoxin blocks fibronectin induction in a dose dependent manner in multiple fibroblast cell lines

**Figure 2:**
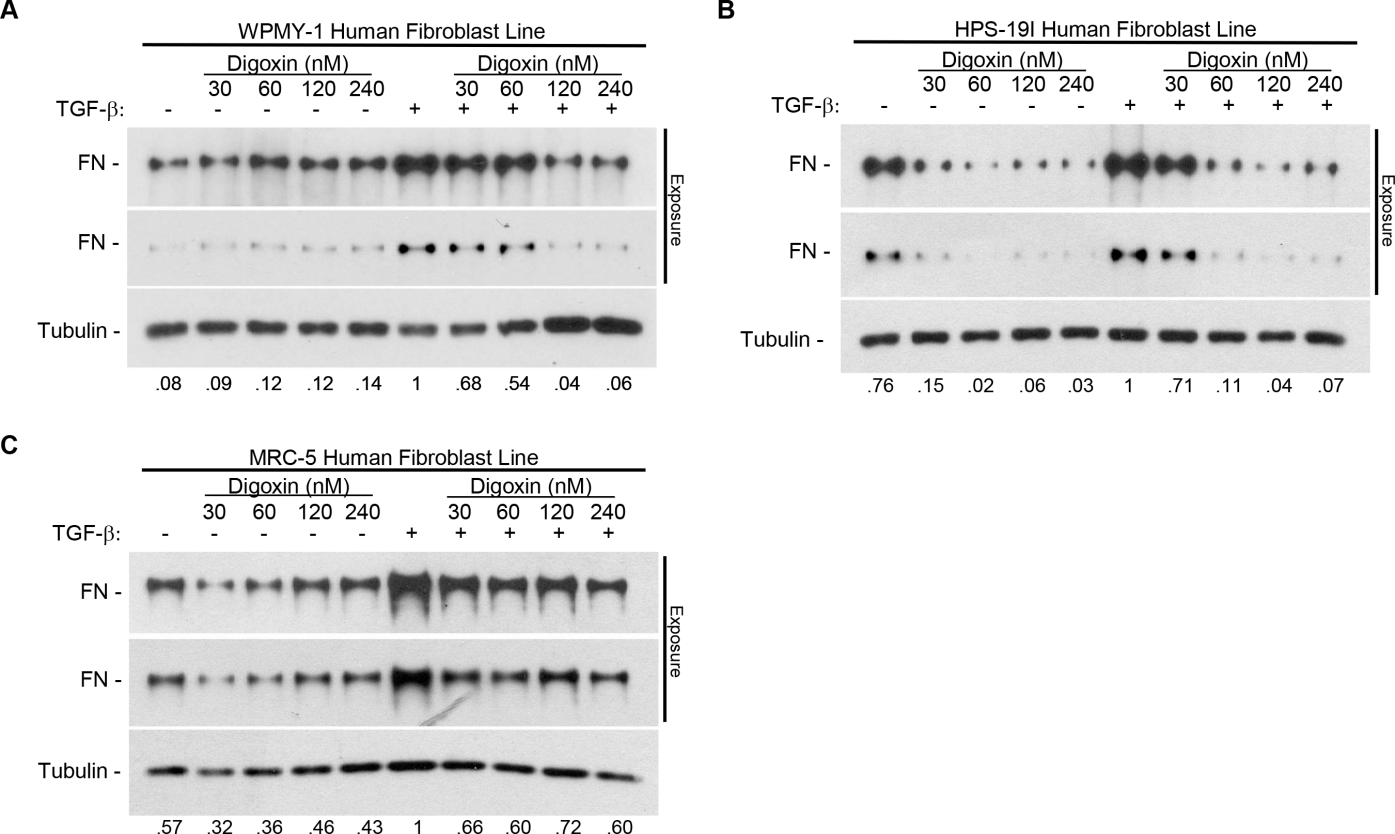
Digoxin prevents TGF-β-induced fibronectin expression in a dose dependent manner in multiple human fibroblast cell lines (**A**) WPMY-1 (**B**) HPS-19I, and (**C**) MRC-5 human fibroblast cells were treated with or without 5 ng/ml TGF-β in the presence or absence of increasing concentrations (30, 60, 120, or 240 nM) of digoxin for 24 hours. Representative blots are shown with two exposures of fibronectin to account for strong signal intensity. Relative densitometry normalized to load control is indicated for each blot.

To determine whether the ability of cardiac glycosides to block fibronectin induction was unique to the WPMY-1 cell line we additionally tested the MRC-5 and HPS-19I lines. MRC-5 fibroblasts were derived from normal lung tissue and HPS-19I from normal prostate [31, 32]. These cell lines were treated with increasing concentrations of digoxin in the presence or absence of 5 ng/ml TGF-β for 24 hours under serum-free conditions. By western blot analysis of whole cell lysates, digoxin was determined to effectively block TGF-β-induced fibronectin induction in each of the three cell lines (Figure 2A–2C). Moreover, it is apparent from these results that digoxin reduces the basal level of fibronectin expression to some extent in MRC-5 and HPS-19I, in particular (Figure 2B, 2C). Together these data suggest the effect of cardiac glycosides on TGF-β-induced fibronectin induction is common to fibroblasts in general.

### Digoxin is able to block TGF-β-induced CAF differentiation

**Figure 3:**
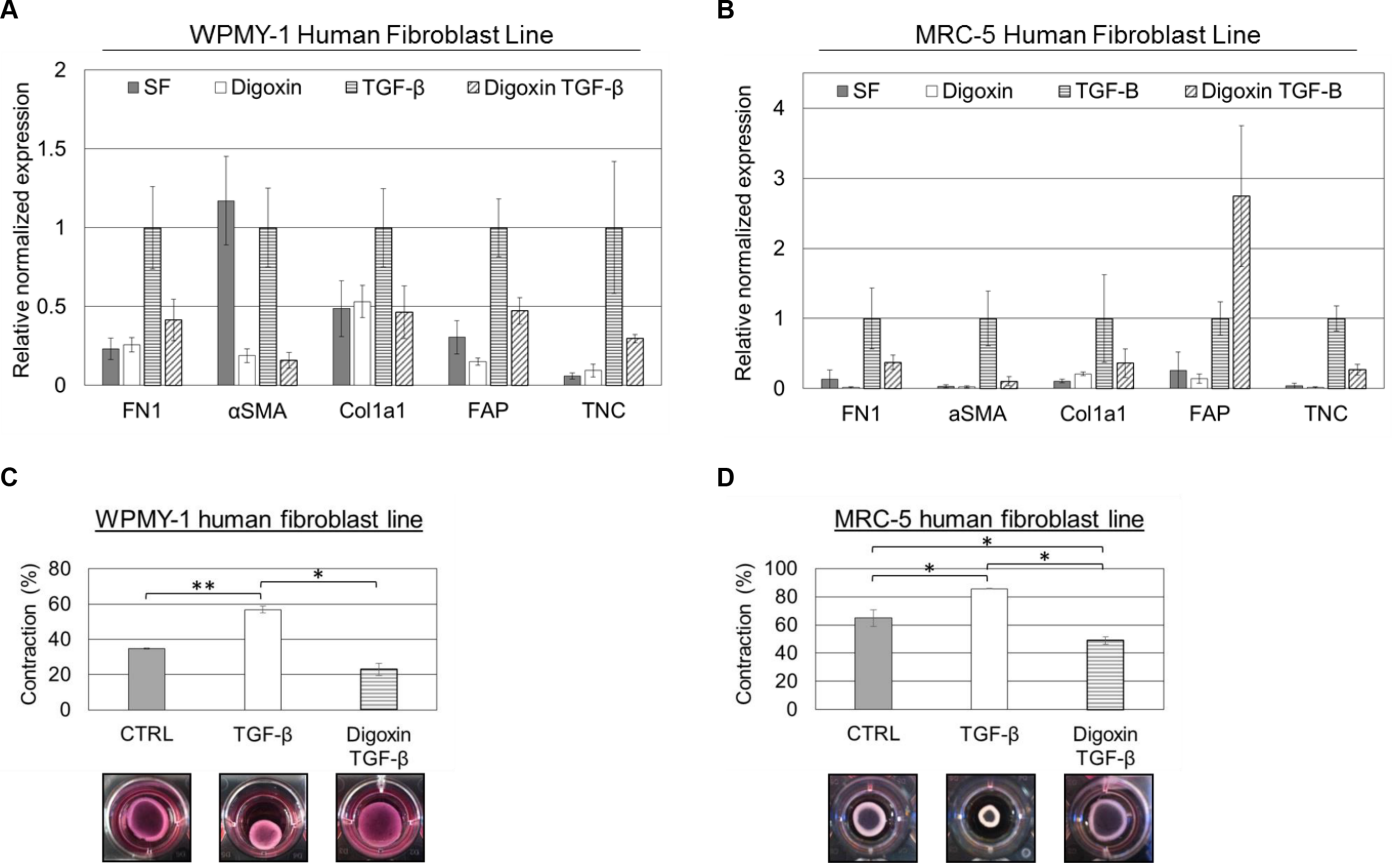
Cardiac glycosides inhibit TGF-β-induced cancer-associated fibroblast (CAF) differentiation in both WPMY-1 and MRC-5 fibroblasts WPMY-1 (**A**) and MRC-5 (**B**) fibroblasts were treated with or without 120 nM digoxin ± 5 ng/ml TGF-β. RNA was isolated from cells 24 hours after treatment. Real-time PCR analysis was performed for the indicated mRNA (*n* = 3, ± SEM). WPMY-1 (**C**) and MRC-5 (**D**) fibroblasts embedded in collagen/Matrigel matrices were treated with or without 120 nM digoxin ± 5 ng/ml TGF-β for 4 days post seeding. Data are shown as percent contracted area from initial 100% well area. *P < 0.05 and **P < 0.01 (*n* = 3, ± SEM). Representative matrices are shown.

Given that cardiac glycosides were able to block TGF-β-induced fibronectin expression in multiple fibroblast lines, we sought to determine whether CAF differentiation in general was prevented. CAF differentiation is associated with gene expression changes to a number of common markers including fibronectin (FN1; Entrez Gene: 2335), α-smooth muscle actin (αSMA; Entrez Gene: 59), collagen 1a1 (Col1a1; Entrez Gene: 1277), fibroblast activation protein (FAP; Entrez Gene: 2191), and tenascin C (TNC; Entrez Gene: 3371) among others. Accordingly, we treated WPMY-1 and MRC-5 cells with 120 nM digoxin, an effective dose based on immunofluorescence and western blot assays, in the presence or absence of 5 ng/ml TGF-β for 24 hours under serum-free conditions. Quantitative real-time PCR was performed on these samples to evaluate gene expression changes to FN1, αSMA, Col1a1, FAP, and TNC. Although cell line specific distinctions were apparent, in general, TGF-β upregulated expression of each of the CAF markers and digoxin was able to block this induction or even reduce the basal level of marker expression (Figure 3A, 3B). In WPMY-1 cells, αSMA expression was exceptionally high under basal conditions, but was greatly reduced with the addition of digoxin. Additionally, FAP expression in the MRC-5 cell line was an anomaly with digoxin causing it to increase when combined with TGF-β.

To further evaluate the ability of cardiac glycosides to prevent CAF differentiation, we tested whether digoxin was able to block the increased contractility characteristic of CAFs. Either WPMY-1 cells or MRC-5 cells were embedded in a matrix of Matrigel and collagen forming a disc across wells of a 24-well plate. After 24 hours, cells in the matrix were treated with TGF-β with or without 120 nM digoxin for 96 hours. Images of the matrix disks were taken after 96 hours of contraction and the area of each was quantitated. Digoxin was able to significantly reduce the ability of both WPMY-1 and MRC-5 fibroblasts to contract the extracellular matrix discs, indicative of blocked CAF differentiation (Figure 3C, 3D). Taken together, these data demonstrate that digoxin is able to prevent multiple characteristic changes of CAF differentiation elicited by TGF-β.

### Digoxin blocks TGF-β-induced SMAD promoter activity likely through Na^+^/K^+^ ATPase inhibition

**Figure 4:**
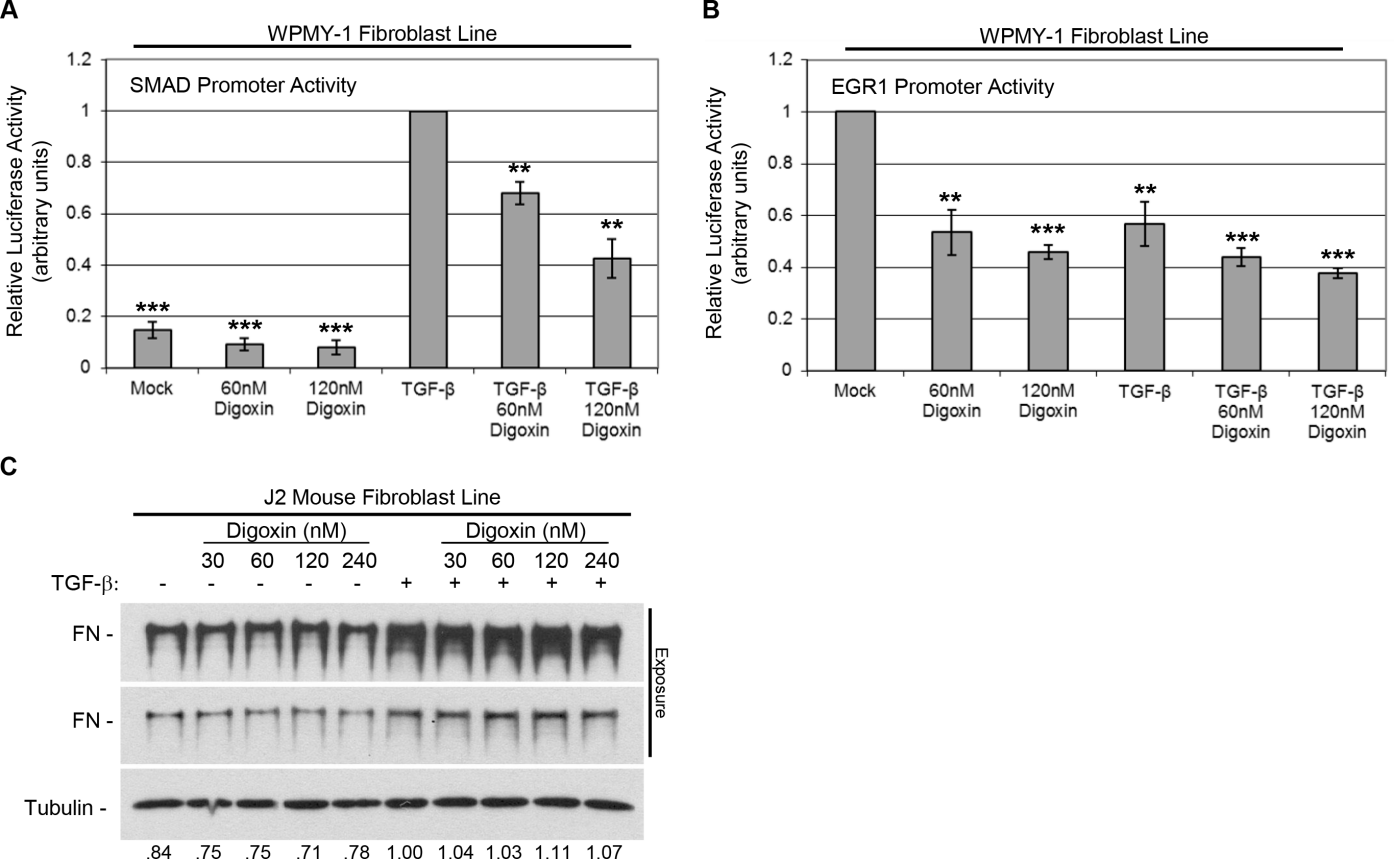
Digoxin prevents TGF-β-induced SMAD promoter activity, but does not prevent TGF-β-induced fibronectin expression in the context of the mouse Na^+^/K^+^ ATPase (**A**, **B**) WPMY-1 human fibroblast cells transfected with SMAD (A) or EGR1 (B) luciferase reporter were treated with or without 5 ng/ml TGF-β in the presence or absence of digoxin (60 or 120 nM) for 24 hours. Relative luciferase activity is shown. *P < 0.05, **P < 0.01, ***P < 0.001 are significant differences compared to values set to 1 (*n* = 3, ± SEM). (**C**) J2 mouse fibroblast cells were treated with or without 5 ng/ml TGF-β in the presence or absence of increasing concentrations (30, 60, 120, or 240 nM) of digoxin for 24 hours. Representative blot is shown with two exposures of fibronectin to account for strong signal intensity. Relative densitometry normalized to load control is shown.

Given that digoxin was able to block global CAF changes responsive to TGF-β, we sought to test whether digoxin impaired TGF-β-induced transcriptional regulation. To this end, we performed luciferase promoter assays for two transcription factors downstream of TGF-β signaling, SMAD 2/3 (Entrez Genes: 4087/4088) and EGR1 (Entrez Gene: 1958), in WPMY-1 fibroblasts after 24 hours of treatment with digoxin ± TGF-β [20, 33, 34]. As expected, TGF-β caused a marked increase in SMAD promoter activity. Digoxin was able to reduce this activity in a dose dependent manner (Figure 4A). Conversely, EGR1 promoter activity in WPMY-1 cells was reduced by TGF-β as well as digoxin treatment, a trend unlikely to contribute to the effect of digoxin on CAF differentiation (Figure 4B).

We next sought to assess whether digoxin prevented CAF differentiation through its cognate target of inhibition, the Na^+^/K^+^ ATPase (Entrez Gene: 476), which would elevate intracellular calcium concentration. It is well established that the affinity of digoxin for the mouse Na^+^/K^+^ ATPase is 10^3^-fold less than that for the human protein [35]. Accordingly, we tested the efficacy of digoxin to block fibronectin induction in the J2 mouse fibroblast cell line. J2 cells were treated with increasing concentrations of digoxin in the presence or absence of TGF-β for 24 hours under serum-free conditions. Digoxin was ineffective in the context of the mouse Na^+^/K^+^ ATPase. TGF-β caused a modest induction of fibronectin over basal levels, but even at the highest concentration, digoxin was not able to reduce fibronectin levels (Figure 4C). These data suggest digoxin disrupts TGF-β-induced SMAD promoter activity by inhibiting Na^+^/K^+^ ATPase-dependent Ca^2+^ signaling.

## DISCUSSION

This study demonstrates for the first time that the cardiac glycoside class of drugs, particularly digoxin, are able to block TGF-β-induced differentiation of fibroblasts to the activated CAF phenotype. Using an immunofluorescence-based screen of repurposed drug libraries to assess fibronectin expression, we found this class of drugs to be uniquely effective. Follow-up studies concluded digoxin blocked fibronectin induction in multiple fibroblast cell lines at low nanomolar concentrations. This effect was without apparent cellular toxicity or disruption of basal proliferation. By assessing the expression of CAF markers and ECM contractility in multiple fibroblast cell lines, we determined digoxin inhibited the activated CAF phenotype induced by TGF-β treatment.

Digoxin was found to reduce activity at the SMAD promoter as a likely explanation for the restricted CAF phenotype. Although some evidence suggests the transcription factor EGR1 can regulate calcium homeostasis and is upregulated downstream of TGF-β signaling, under our experimental conditions, the changes to EGR1 expression would not account for the effect of digoxin on CAF differentiation [34, 36]. Given that digoxin is largely ineffective in a mouse fibroblast line, it is likely that digoxin works through its known target of inhibition, the Na^+^/K^+^ ATPase [35]. This conclusion is further supported by the fact that all represented cardiac glycosides in the library screens were identified as potent hits (roughly one-third of the 27 total hits from the 1731 compounds screened), highlighting the importance of calcium signaling downstream of TGF-β in CAF activation. Although there are multiple reports of calcium signaling regulating SMAD activity through direct phosphorylation by Ca^2+^/calmodulin-dependent protein kinase II (CAMKII), the effect seems highly context dependent. Whereas CAMKII activity promotes SMAD1 transcriptional activity in undifferentiated mesenchymal cells, CAMKII inactivates SMAD2 in fibroblast-like Cos-1 cells [34, 37, 38].

To evaluate the effects of digoxin on the overall CAF phenotype, we assessed changes in gene expression of known CAF markers. These markers however, are also functionally relevant to the progression of cancer, in and of themselves [39]. Fibronectin and collagen I can initiate invasive cell signaling pathways through integrin signaling [40–44]. Elevated levels of the extracellular glycoprotein tenascin C correlate with poor prognosis in multiple cancer types including breast and bladder cancer as well as being associated with increased invasiveness [45]. α-smooth muscle actin is a critical mediator of the contractile apparatus in myofibroblasts and is therefore directly related to ECM remodeling [46]. Inhibition of the serine protease fibroblast activation protein has been shown to reduce growth in numerous mouse tumor models [47, 48]. Digoxin was found to repress the expression of each marker downstream of TGF-β.

In addition to gene expression changes, we found that digoxin was able to prevent the enhanced contractile phenotype of CAFs. Normally, enhanced contractility of myofibroblasts is essential to the ECM reassembly required for wound healing [49]. In addition to ECM remodeling, constriction within a tissue microenvironment influences cells through mechanotransduction to elicit a motile or invasive phenotype [50]. In the context of cancer, unresolved stromal rigidity imparts pro-invasive signals to the progressing tumor while disrupting normal restrictive tissue architecture [51]. A number of studies have attributed tissue stiffness to tumor aggressiveness and promotion of metastasis [52]. The ability of digoxin to prevent this phenotype supports its evaluation as a therapeutic strategy for normalizing the tumor microenvironment [53].

Although cardiac glycosides have special toxicity considerations in patients, over a million patients in the United States are receiving digoxin for heart failure [29, 54]. To our knowledge, there are no reports specifically demonstrating the ability of cardiac glycosides to prevent CAF differentiation. Interestingly, recently published data indicate an anti-fibrotic effect of ouabain through upregulation of COX-2 in lung fibroblasts, likely sharing a mechanism with our findings at the level of SMAD activity [55]. Given the abundance of published reports demonstrating that CAFs are able to facilitate the invasiveness of otherwise quiescent tumor cells, work toward the development of CAF-targeting therapeutic agents is warranted [8]. Although some drugs against stromal targets, such as angiogenesis inhibitors, have been developed, options for targeting fibroblast activation are greatly limited. Sibrotuzumab, an antibody to FAP, has been shown to target colorectal and non-small cell lung cancer tumor sites in a phase I trial, but data on efficacy is limited [56]. Our findings highlight that the Na^+^/K^+^ ATPase, as a target, and cardiac glycosides, as agents, may be a fruitful area of research for the development of novel therapeutics aimed at restricting fibroblast activation and the CAF phenotype. These drugs may be particularly useful in prostate and pancreatic cancer where the reactive stroma, consisting of CAFs, is a major factor in tumor progression and metastasis.

## MATERIALS AND METHODS

### Cell Culture

The WPMY-1 prostate fibroblast cell line was obtained from ATCC (CRL-2854) and maintained in DMEM (Corning, Manassas, VA) 5% fetal bovine serum (FBS) (Gemini, West Sacramento, CA). WPMY-1 cells were derived from the stroma adjacent to a normal adult prostate. The MRC-5 lung fibroblast cell line was obtained from ATCC (CCL-171) and maintained in EMEM (Corning) supplemented with 10% FBS. MRC-5 cells were derived from normal fetal lung tissue. These cells were authenticated by Promega through short tandem repeat analysis. HPS-19I prostate fibroblast cells were obtained from Dr. David Rowley (Baylor College of Medicine) and were recently analyzed by spectral karyotyping. These cells were maintained in DMEM with 5% FBS, 5% Nu-Serum (Gemini), 5 μg/ml insulin, 0.5 μg/ml testosterone, 25 μg/ml penicillin-streptomycin. 3T3-J2 (J2) embryonic mouse fibroblasts were obtained from Dr. Jason Bodily and grown in DMEM with 10% bovine serum (not fetal). All cells were maintained at 37°C, 5% CO_2_.

### Reagents

Recombinant TGF-β protein, digoxin (≥ 95%), g-strophanthin (ouabain, ≥ 95%), and lanatoside C (≥ 95%) were purchased from Sigma-Aldrich.

### Cellomics high content screening

The 1280 compound Prestwick Chemical Library (Prestwick Chemical, Illkirch-Graffenstaden, France) and the 451 compound NIH Clinical Collection (Evotec, Hamburg, Germany) consist exclusively of FDA approved compounds. All compounds were provided at a concentration of 10 mM in DMSO. Cells were seeded at 7 × 10^3^ cells per well in a 96-well plate. The following day, media was changed to serum-free DMEM for 24 hours. After serum-starvation, cells were treated with 5 ng/ml TGF-β and 10 μM (final DMSO concentration of < 0.1%) of each screening compound for 24 hours. The solvent, 0.1% DMSO ± TGF-β served as an internal control on each plate to normalize day-to-day and plate-to-plate variation. Cells were then fixed with 100% methanol for 10 minutes at −20°C, washed, and stained with anti-fibronectin (F3648, 1:200 dilution) (Sigma-Aldrich) in a solution of 0.25% bovine serum albumin, 0.1% saponin, and PBS (termed BSP) at 4°C overnight. The following day, cells were washed and incubated with secondary 594-conjugated antibody (1:200) (Jackson ImmunoResearch, West Grove, PA) in BSP for 1 hour at room temperature. Cells were washed and stained with SlowFade Gold reagent with DAPI (S36938) (Invitrogen, Carlsbad, CA) for 15 minutes and washed prior to visualization. Cells were visualized and data collected using the Cellomics High Content Screening platform (Thermo Scientific, Waltham, MA). Cellomics software analyzed intensity of fibronectin staining within a ring 6 to 12 pixels from the nucleus for at least 5 fields per well. Compounds that decreased the intensity of fibronectin staining below levels of the positive control (TGF-β alone) and produced a response in a dose-dependent manner were selected for further study.

### Quantitative real-time PCR

Cells were seeded at 10^6^ cells per 10 cm dish and were allowed to grow to 80% confluency in complete media. Cells were removed with 0.025% EDTA and centrifuged for 5 minutes at 1100 rpm. Cell pellets were resuspended in 1 ml Trizol (Life Technologies, Carlsbad, CA) and RNA was extracted according to the manufacturer's protocol. The SuperScript First-Strand kit (Life Technologies) was used to synthesize cDNA from 5 μg total RNA. Quantitative PCR was set-up using RT^2^ SYBR Green Flour FAST Mastermix (Qiagen, Venlo, Netherlands) and run on a Bio-Rad CFX96 Real-Time PCR Detection System (Bio-Rad, Hercules, CA). Data were analyzed using Bio-Rad CFX Manager 3.0 software and are shown as relative fold change. For all PCR reactions, GAPDH was used as an endogenous control and CT values were normalized to levels of GAPDH expression. Primers were designed using Integrated DNA Technologies PrimerQuest software. Sequences used to analyze RNA expression include: FN1 Forward: 5′- CTGAGACCACCATCACCATTAG-3′, Reverse: 5′- GAT GGTTCTCTGGATTGGAGTC-3′; COL1A1 Forward: 5′- CCTGTCTGCTTCCTGTAAACTC-3′ Reverse: 5′- GT TCAGTTTGGGTTGCTTGTC -3′; ACTA2 Forward: 5′- GATGGTGGGAATGGGACAAA-3′, Reverse: 5′- GCCA TGTTCTATCGGGTACTTC-3′; FAP Forward: 5′- TGAGC TTCCTCGTCCAATTC-3′, Reverse: 5′-GTGGATCTCC TGGTCTTTGTT-3′; TNC Forward: 5′- CTCTGGCCTCT ACACCATTTATC-3′, Reverse: 5′- TGCGTCTCAGGAAC ACAATC-3′; GAPDH Forward: 5′- CAAGAGCACAAGA GGAAGAGAG-3′, Reverse: 5′- CTACATGGCAACTGT GAGGAG-3′. Results are averaged from three independent experiments.

### Viability assay

Cells were plated at 20% confluency and allowed to grow in complete media 1 day prior to treatment with each cardiac glycoside at their respective IC_50_ of fibronectin inhibition for 24 and 48 hours in DMEM containing 1% FBS. Cell viability was assessed using the Cell Titer Blue Cell Viability assay (Promega, Madison, WI) according to manufacturer's protocol. Data were acquired using the Synergy4 multi-detection plate reader (BioTek, Winooski, VT), measuring fluorescence at 560/590 nm. The assay was performed three times with 12 samples per treatment.

### Western blot analysis

Cells were seeded at 70% confluency in 24- well plates. Following treatments, lysates were taken in boiling Laemmli buffer (125 mM Tris, 4% SDS, 0.01% bromophenol blue, 30% sucrose) with 0.05% β-mercaptoethanol (BME) and boiled for 5 minutes. Samples were analyzed by SDS-PAGE and blotted with the indicated primary antibodies: fibronectin (H-300, 1:2000 dilution; Santa Cruz) and β-tubulin (1:5000; Neomarkers, Fremont, CA). Blots were subsequently probed with horseradish peroxidase-conjugated secondary antibodies (1:5000) (Amersham Biosciences, Pittsburgh, PA), and detection was acquired with Pierce ECL 2 (Thermo Fischer Scientific). Representative blots were cropped and enhanced with Adobe Photoshop 7.0. All western blotting experiments were performed at least three times.

### Ecm contractility assay

The methods for this assay were a variation of the assay performed by Calvo *et al.* [51]. Briefly, 5 × 10^5^ cells per well were mixed with a final concentration of 4.6 mg/ml rat tail collagen I and 2.2 mg/ml Matrigel (BD Biosciences). Each well of a 24-well plate was covered with 300 μl of the matrix/cell suspension and allowed to polymerize for 1 hour at 37°C. After 1 hour, the matrix was hydrated with 500 μl complete media at 37°C overnight. The gels were detached from the sides of the tissue culture plastic with a spatula, treated with or without 5 ng/ml TGF-β ± digoxin, and allowed to contract for 4 days. The wells were treated with fresh media and treatments on day 2 post seeding. Images were analyzed using ImageJ software to determine gel area relative to the well. The assays were performed three times in duplicate.

### Luciferase reporter assay

SMAD (CCS-017) and EGR1 (CCS-8021)-responsive luciferase constructs were used according to the manufacturer's instructions (Qiagen). In short, manufacturer-engineered constructs were transfected into WPMY-1 cells for 24 hours. Transfected cells were treated with TGF-β ± digoxin for an additional 24 hours. Luciferase activity was measured using the Promega Dual-Luciferase Reporter Assay System, with *Renilla* luciferase as an internal control. Experiments were performed three times in duplicate.

### Statistical analysis

Data are expressed as means ± SEM. Statistical analysis was performed using Student's *t*-test (two tailed) with *P*-value < 0.05 as significant.
